# Navigating Hope and Illness Cognition in Advanced Ovarian Cancer Patients: A CSM‐Based Phenomenological Study

**DOI:** 10.1002/pon.70506

**Published:** 2026-06-12

**Authors:** Yun Long, Chunchang Zhong, Cuili Wen, Fang He, Yufen Wang, Cairong Wang, Hui Xia, Yunhuang Shi, Ping Jin

**Affiliations:** ^1^ Department of Gynecology Shenzhen Maternity and Child Healthcare Hospital Women and Children's Medical Center Southern Medical University Shenzhen China

**Keywords:** advanced ovarian cancer, hope, illness cognition, qualitative research, therapeutic alliance

## Abstract

**Background:**

Advanced ovarian cancer patients face profound psychosocial challenges in maintaining hope amidst terminal illness and treatment uncertainty. Understanding mechanisms supporting adaptive hope could inform psychosocial interventions.

**Methods:**

Phenomenological qualitative study of 16 women with advanced ovarian cancer receiving platinum‐based and/or targeted therapies at a tertiary center in southern China. Semi‐structured interviews conducted across 11‐month observation period; thematic analysis guided by Leventhal’s Common‐Sense Model (CSM).

**Results:**

Participants demonstrated a triphasic psychosocial adjustment process organized around three interdependent themes: (1) Cognitive Reappraisal of Chronicity: Patients progressively reconceptualized ovarian cancer from acute crisis to manageable chronic condition, anchored in biomedical evidence (stable disease scans) and analogized to familiar chronic illnesses (diabetes). This cognitive restructuring enabled milestone‐based temporal orientation replacing survival countdown framing; (2) Therapeutic Alliance as Hope Anchor: Quality of patient–clinician relationships functioned as relational scaffolding enabling cognitive reappraisal through shared decision‐making, emotional attunement, continuity of care, and hope‐framed honest prognostic communication. Family members facilitated this process through co‐construction of illness identity, milestone tracking, and selective information mediation (81.3% of participants); (3) Strategic Information Management: Patients actively regulated illness‐related information engagement, prioritizing actionable biomarkers over distressing epidemiological statistics, protecting the chronic illness cognitive framework while maintaining decision‐making capacity. The triphasic trajectory progressed from Phase 1 Crisis Cognition (0–3 months) through Phase 2 Cognitive Negotiation (4–10 months) to Phase 3 Adaptive Integration (11+ months). Platinum‐resistant cases reverted to Phase 1, indicating dynamic rather than stable cognitive achievement. All themes directly mapped onto CSM regulatory dimensions (identity, timeline, consequences, controllability, emotional representation), demonstrating empirical alignment between data‐derived constructs and established theoretical architecture.

**Conclusions:**

Hope maintenance in advanced ovarian cancer depends on integrated cognitive reappraisal, relational security, and behavioral information management—mechanisms actionable through targeted psychosocial intervention. Family‐centered communication and milestone‐based temporal scaffolding warrant clinical implementation pending prospective validation.

## 1 Background

Advanced ovarian cancer treatment has undergone considerable change with precision medicine approaches including PARP inhibitors and anti‐angiogenic agents [1]. Landmark trials such as SOLO‐1 demonstrate meaningful progression‐free survival improvements (HR 0.30, 95% CI 0.23–0.41) with olaparib maintenance therapy, particularly in BRCA‐mutated cases [2]. While these advances extend survival, patients face prolonged disease trajectories marked by cumulative treatment toxicities and psychological distress [3]—highlighting the importance of understanding how patients cognitively frame their illness experience amidst evolving therapeutic possibilities.

Leventhal's Common‐Sense Model (CSM) offers a cognitive‐emotional framework for understanding illness perception and coping [4, 5]. Originally proposed by Leventhal, Meyer, and Nerenz [6], the model posits that individuals construct lay representations of illness along five core dimensions: identity, timeline, consequences, cause, and controllability/curability. These representations guide coping strategies and influence health outcomes [4, 5]. Meta‐analytic evidence indicates illness representations systematically associate with coping and outcomes: greater perceived controllability relates to adaptive coping, while higher symptom identity and negative consequence appraisals associate with psychological distress [7]. In cancer populations, maladaptive representations—particularly high symptom identity and negative consequence perception—associate with anxiety, depression, and reduced quality of life [8]. CSM‐based interventions improve symptom management and quality of life [9].

Advanced ovarian cancer presents a distinctive CSM context: high recurrence rates, complex long‐term regimens, and substantial psychological burden [10, 11]. PARP inhibitor maintenance therapy creates sustained demands on illness appraisal. Emerging evidence links maladaptive illness representations to 42% non‐adherence to PARP inhibitors [12], indicating clinical relevance of cognitive processes, though causality remains undetermined.

Despite CSM's oncology utility, ovarian cancer application remains limited. Mechanisms through which illness identity construction interacts with hope maintenance under therapeutic uncertainty require deeper examination [4]. How therapeutic alliance and mechanistic understanding of PARP inhibitors shape internal controllability representations remains largely unexplored.

Hope encompasses goal‐directed agency and pathway thinking [13], serving as a vital psychological resource in chronic oncology care. Higher hope levels associate with significantly improved treatment adherence, enhanced quality of life, and favorable survival outcomes [14]. However, in ovarian cancer—characterized by late diagnosis (60% Stage III‐IV at presentation) and high recurrence rates [15]—hope's protective benefits exist in dynamic tension with risk of unrealistic therapeutic expectations.

Disease cognition, comprising illness representations and self‐regulatory processes, constitutes the interpretative lens through which patients derive meaning from cancer experience. Accurate illness perceptions correlate with reduced psychological distress and better outcomes [16, 17]. Yet ovarian cancer patients face unique cognitive challenges [14]. The CSM framework maps directly onto cognitive‐emotional paradoxes in ovarian cancer—for instance, why patients oscillate between viewing their condition as acute versus chronic [18].

Critical literature gaps exist: Although hope and cognition have each been investigated in cancer populations, their interactive relationship in ovarian cancer remains poorly understood. In addition, prior research has largely relied on quantitative approaches, offering limited insight into the lived and meaning‐making processes through which patients interpret their illness [19]. This gap is further compounded by the relative neglect of cultural influences, despite evidence that illness perceptions are deeply embedded in sociocultural contexts, particularly in Chinese healthcare settings characterized by family‐centered decision‐making [20]. Given its capacity to accommodate cultural variation, the CSM provides a useful framework for addressing these gaps [4].

This study examines how CSM‐based illness representations relate to hope and treatment adherence among ovarian cancer patients receiving maintenance therapy, with particular attention to cognitive‐emotional processes mediating psychosocial adjustment.

### 1.1 Aim

The main aim of this study is to explore the interplay between illness cognition and hope maintenance in advanced ovarian cancer patients through a phenomenological lens, specifically examining how patients' cognitive reappraisal of disease chronicity shapes hope trajectories and informs the development of targeted psychosocial interventions in Chinese healthcare contexts.

## 2 Materials and Methods

### 2.1 Description of the Study Area

This study was conducted at Shenzhen Maternity and Child Healthcare Hospital, Women and Children's Medical Center, Southern Medical University. The hospital serves a diverse population of over 1.5 million annual outpatients and 50,000 inpatients from across China and internationally. The Gynecology Department, where this research was based, maintains 231 beds and performs more than 10,000 surgical procedures annually. Approximately 200 patients with gynecologic cancers are treated annually, including more than 100 patients with ovarian cancer. Our institution possesses robust clinical experience and sufficient patient volume to support this investigation.

### 2.2 Study Approach and Period

This qualitative study adopted an Interpretative Phenomenological Analysis (IPA) framework [21] to understand participants' individual experiences and subjective meaning construction regarding illness cognition. This approach was selected for its capacity to honor particularity of lived experience while remaining sensitive to psychological and contextual dimensions of chronic illness management—especially pertinent to a population navigating existential uncertainties of advanced ovarian cancer. The study adhered to the Consolidated Criteria for Reporting Qualitative Research (COREQ) [22] to ensure quality and transparency. Data were collected between November 2023 and September 2024.

### 2.3 Participants and Eligibility

This study recruited 16 advanced ovarian cancer patients (FIGO Stage III–IV) from Shenzhen Maternity and Child Healthcare Hospital, Women and Children's Medical Center, Southern Medical University, through purposive sampling between November 2023 and September 2024.

#### 2.3.1 Screening and Enrollment

From 142 potential participants assessed for eligibility, 126 were excluded: failure to meet histological and staging criteria (*n* = 76), declining participation (*n* = 28), loss to follow‐up prior to enrollment (*n* = 12), or failure to meet baseline assessment thresholds (*n* = 10).

**FIGURE 1:**
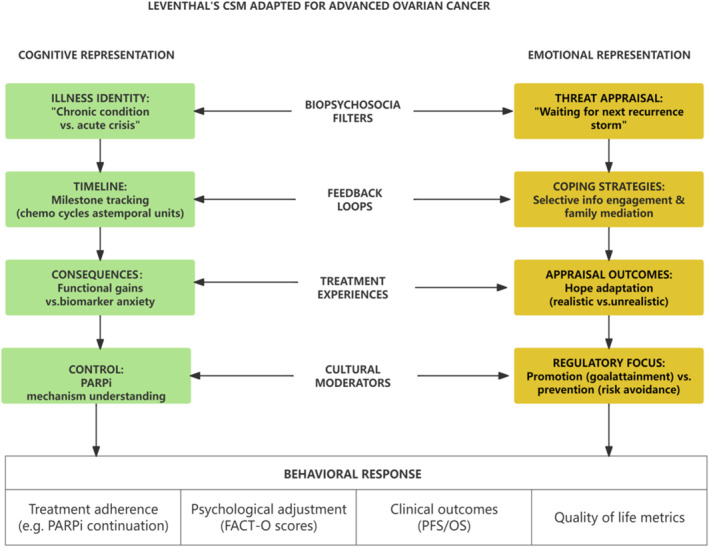
Leventhal's CSM adapted for advanced ovarian cancer.

The 76 exclusions warrant clarification. While approximately 70%–75% of epithelial ovarian cancer diagnoses occur at FIGO Stage III–IV [23], the recruitment pool was drawn from a tertiary‐level women's and children's medical center—not a dedicated oncology center. This institution receives substantial referral volume for early‐stage gynecologic cancers from regional screening programs, non‐epithelial ovarian tumors (germ cell, sex cord–stromal, borderline/low malignant potential tumors), non‐ovarian primary gynecologic malignancies, and patients not meeting the specific PARP inhibitor maintenance therapy criterion. The elevated exclusion rate reflects this sampling frame effect rather than an anomalous stage distribution in the underlying ovarian cancer population. The complete screening and enrollment flow is presented in Figure 1 (CONSORT‐adapted diagram).

#### 2.3.2 Inclusion and Exclusion Criteria

##### 2.3.2.1 Inclusion Criteria

① Histologically confirmed epithelial ovarian cancer, FIGO Stage III–IV; ② Completion of ≥ 6 cycles of platinum‐based chemotherapy; ③ Currently receiving or having completed PARP inhibitor and/or immunotherapy maintenance; ④ Adequate expressive and communicative capacity for in‐depth qualitative interview participation; ⑤ Voluntary informed consent and willingness to articulate treatment experiences.

##### 2.3.2.2 Exclusion Criteria

① Severe cognitive impairment or active psychiatric comorbidity; ② Estimated life expectancy < 6 months (palliative‐only care pathway).

#### 2.3.3 Histological Subtype Composition

All 16 participants carried a confirmed diagnosis of high‐grade serous ovarian carcinoma (HGSOC) [24], the predominant histological subtype and primary PARP inhibitor–indicated histology in current clinical practice. While subtype homogeneity enhances within‐group comparability, it limits generalizability to other histological subtypes: ① Clear cell carcinoma: Characterized by relative platinum resistance, which may impede the cognitive reframing of cancer as a “manageable chronic condition” central to this study's adjustment process; ② Low‐grade serous carcinoma (LGSOC): Marked by poor chemotherapy responsiveness; its indolent yet treatment‐resistant trajectory may generate qualitatively different patterns of temporal orientation and hope maintenance not captured by the current model; ③ Mucinous carcinoma: A rare subtype requiring atypical treatment protocols, in which illness identity construction and help‐seeking behaviors may differ substantially from established PARP inhibitor care pathways. Future research should employ deliberate histological sampling strategies—including purposive recruitment of clear cell, LGSOC, and mucinous subtype patients—to examine whether this CSM‐grounded illness cognition framework is applicable to the broader ovarian cancer population.

### 2.4 Sampling Technique and Sample Size Rationale

This study implemented phenomenological purposive sampling to recruit participants capable of providing rich experiential data on illness cognition and hope maintenance during PARP inhibitor maintenance therapy. Purposive sampling was selected because the study aimed at theoretical elaboration of the CSM framework rather than population‐level prevalence estimation [21].

Sample size was determined by iterative thematic saturation, a standard criterion for qualitative adequacy in IPA research [21]. Primary theme saturation was reached at interview 14 and confirmed through two additional cases (*n* = 15, 16) with no new CSM‐relevant categories emerging. The final sample of 16 is consistent with published guidance for longitudinal IPA studies of homogeneous clinical populations, recommending 9–17 participants for adequate depth and transferability [25].

Demographic and clinical diversity was deliberately maintained across: age range 38–72 years; treatment duration 4–28 months; BRCA1/2 mutation status; and PARP inhibitor regimen (olaparib, niraparib). This diversity captured variation in illness cognition across the disease maintenance trajectory rather than achieving statistical representativeness.

The 11.3% enrollment rate (16/142) reflects cumulative effects of multi‐criterion eligibility filtering, voluntary participation requirements, and purposive sampling design.

### 2.5 Interview Instrument Development

#### 2.5.1 Theoretical Framework and Initial Guide Construction

Semi‐structured interview guide developed through four‐phase iterative process anchored in Leventhal's Common Sense Model (CSM) [6]. Initial questions mapped onto five CSM dimensions:

Illness Identity: How would you explain your condition to someone unfamiliar with ovarian cancer?

Timeline: What does “long‐term survival” mean in your daily life?

Consequences: How has this illness redefined your priorities?

Control/Cure: What helps you feel most empowered during treatment?

Emotional Response: Can you describe a moment when fear and hope coexisted?

Two clinical psychologists and one medical anthropologist achieved consensus following two revision rounds.

#### 2.5.2 Pilot Testing and Iterative Refinement

Three pilot interviews identified three critical adaptations: ①Terminology barriers: The term “biomarker” was replaced with “blood test results” after all three pilot participants indicated unfamiliarity. ②Emotional safeguards: Buffer questions were inserted before recurrence‐related probes (e.g., “Tell me about a recent good day”) to reduce acute distress responses. ③Cultural contextualization: Family‐centric decision‐making probes were incorporated (e.g., “Who helps you interpret scan results?”), consistent with documented patterns of family information mediation in Chinese oncology contexts [20].

#### 2.5.3 Expert Consensus Validation

Five specialists (two psycho‐oncologists, two nurse researchers, and one qualitative methodologist) assessed item relevance on a 5‐point scale. Illness Identity demonstrated the highest mean relevance score (4.8), prompting the addition of example prompts to improve clarity. The Control/Cure dimension achieved a mean score of 4.1, and compound questions were subsequently split into two separate items. The Emotional Response dimension received a mean score of 3.9; therefore, the term “depression” was removed, as it had been identified in pilot testing as culturally stigmatizing.

#### 2.5.4 Final Interview Guide Structure and Core Questions

Six core questions organized across three domains with integrated visual supports:

*Domain 1: Illness Representations* (with color‐coded symptom cards)

Q1 “How do you view your illness?”
Q2 “How does disease affect life values and lifestyle?”

*Domain 2: Hope Dynamics* (with hope trajectory drawing tool [26])

Q3 “How do you cope with clinical decisions?”

*Domain 3: Therapeutic Alliance* (with family sub‐probes)

Q4 “What personal and social resources help you?”
Q5 “What would you have liked staff to do differently?”
Q6 “How does disease affect social interactions?” [Family probe: “Who helps interpret scans?” [20]]

#### 2.5.5 Cultural and Linguistic Adaptation

To ensure culturally appropriate data collection in the Chinese clinical context, the study incorporated adaptations that reflected local relational and linguistic norms. Interview probes included guanxi‐informed relationship items, such as asking participants how they would describe their bond with their oncologist, in order to capture the role of therapeutic alliance within Chinese relational healthcare norms [27]. In addition, all instruments underwent forward and backward translation by bilingual clinical researchers to ensure linguistic accuracy and consistency. This process was further strengthened by a local idiomatic review conducted by a Mandarin‐speaking medical anthropologist to ensure that the wording was culturally appropriate and meaningful within the Chinese clinical setting.

#### 2.5.6 Observational Design and Nature of Described Clinical Practices

Purely observational, longitudinal IPA design. No novel interventions introduced. Therapeutic practices described (prognostic framing, family information mediation, shared decision‐making) represent naturally occurring clinical practices participants experienced as routine care, neither designed nor standardized by research team.

### 2.6 Data Collection and Quality Assurance

#### 2.6.1 Interview Procedures

Between November 2023 and September 2024, a single trained investigator conducted face‐to‐face, semi‐structured interviews with 16 ovarian cancer patients at Shenzhen Maternity and Child Healthcare Hospital in a private consultation room, ensuring a quiet, private, and clinically familiar setting. Pre‐interview briefing covered research objectives, voluntary participation, confidentiality, and withdrawal rights. Written informed consent was obtained before audio recording commenced.

Interviews lasting 30–60 min were conducted in Mandarin by the first author (an experienced qualitative researcher), audio‐recorded with written consent, and transcribed verbatim within 3 days with double‐check for accuracy. The semi‐structured interview guide explored treatment experiences, including questions such as “Can you describe your treatment decision‐making process?” Detailed field notes were maintained to capture non‐verbal communications and contextual observations. Investigator consistency was maintained across all timepoints to support longitudinal rapport and data comparability.

Participant characteristics: Mean age = 52.3 ± 9.1 years; 68.8% platinum‐resistant disease (detailed in Table 1).

**TABLE 1:** Participants' characteristics (*n* = 16).

	*n*	%
Age at diagnosis (years)		
Mean (standard deviation)	47 (1.82)	
Median (range)	48 (38–69)	
Chemotherapy		
Yes	16	100
No	0	0
Lmmunotherapy/targeted therapy		
Yes	9	56.25
No	7	43.75
Surgery		
Yes	10	62.5
No	6	37.5
Education level		
Bachelor's degree or above	3	18.75
Middle school	8	50
Primary school	5	31.25
Work status		
Working	1	6.25
Leave of absence	5	31.25
Unemployed	10	62.5
Monthly per capita household income (RMB)		
< 10,000	3	18.75
10,000–20,000	8	50
> 20,000	5	31.25
Marital status		
Married	13	81.25
Divorced	0	0
Widowed	1	6.25
Single	2	1.25

#### 2.6.2 Quality Assurance in Data Collection

Member checking was performed by returning transcripts to 5 randomly selected participants (31% of the sample) for verification. Prolonged engagement was achieved through repeated interactions with participants during treatment follow‐ups (median 3 contacts per participant). Pilot interviews with three non‐participant ovarian cancer patients from the same institution helped refine questioning techniques prior to formal data collection.

### 2.7 Data Analysis

We employed a hybrid inductive‐deductive analytic approach integrating inductive content analysis with CSM‐informed deductive structuring across three sequential phases.

#### 2.7.1 Phase 1: Open Coding (Inductive)

All transcripts underwent line‐by‐line open coding using inductive content analysis procedures [28], involving: (1) extraction of significant statements reflecting participants' illness experiences; (2) grouping of initial codes into emergent subcategories based on semantic similarity (e.g., “Treatment Efficacy Optimism,” “Existential Anxiety”). Coding proceeded inductively without imposing a priori theoretical categories, preserving phenomenological integrity.

#### 2.7.2 Phase 2: CSM‐Informed Codebook Development and Thematic Organization (Deductive)

Emergent codes and subcategories were systematically mapped to Leventhal's CSM five illness representation dimensions: identity, timeline, consequences, cause, and controllability/emotional response [4]. This deductive layer served three functions:

① *Codebook organization:* Inductively derived codes were organized within a CSM‐structured codebook, with codes assigned to corresponding CSM dimensions (e.g., oscillation between “acute” and “chronic” framing mapped to Identity; scan‐based temporal planning mapped to Timeline; perceived treatment agency mapped to Controllability).
② *Thematic development:* Higher‐order themes integrated inductively generated subcategories with CSM dimensional logic. Van Manen's lifeworld existentials—Temporality and Relationality—provided additional organizational scaffolding for themes spanning multiple CSM dimensions.
③ *Gap identification:* Codes not mapping cleanly onto existing CSM dimensions were treated as theoretically generative, informing identification of novel mechanisms (e.g., strategic information management) and contributing to conceptual extension of the CSM framework.

#### 2.7.3 Phase 3: Validation

Inter‐coder agreement was established through independent coding by two researchers, with discrepancies resolved via consensus discussion involving a third investigator [25]. Thematic saturation was achieved after 14 interviews and confirmed by 2 additional cases with no novel codes emerging. NVivo 16 facilitated codebook management and thematic mapping [29].

### 2.8 Trustworthiness

The trustworthiness of this study was ensured through four established qualitative criteria outlined by Lincoln and Guba [30]. Credibility was established through prolonged engagement with participants, which enabled the researchers to develop a deeper understanding of participants' experiences and perspectives. It was further strengthened through member checking of preliminary findings with five participants, representing 31% of the sample, as well as through weekly peer debriefing sessions among three researchers to verify the interpretive accuracy of emerging themes. In addition, thick description of both verbal accounts and observed behaviors was documented in field notes to support a credible interpretation of the data.

Transferability was facilitated by providing thick, contextually rich descriptions of the study setting, participant characteristics, and methodological procedures. Detailed demographic and clinical documentation also enabled readers to assess the applicability of the findings to comparable contexts.

Dependability was maintained through a comprehensive audit trail that documented all analytical decisions, codebook revisions, and methodological adaptations throughout the research process. Investigator consistency across all timepoints further supported the dependability of the study.

Confirmability was achieved through researcher reflexivity and transparent documentation of analytical assumptions. It was also strengthened through systematic cross‐verification of codes to ensure that the findings reflected participants' accounts rather than researcher predispositions.

### 2.9 Ethical Consideration

Ethical approval was obtained from Shenzhen Maternity and Child Healthcare Hospital, Women and Children's Medical Center, Southern Medical University (SFYLS[2023]039). All participants were informed about the study purpose and content. Written informed consent was obtained prior to in‐depth interviews. Participants were made aware they could withdraw at any time. Personal information and interview content were kept anonymous and confidential. All data were used solely for research purposes with access restricted to the research team only.

All researchers completed CITI certification in qualitative research ethics. Interview transcripts were de‐identified using pseudonymization protocols compliant with GDPR standards.

## 3 Results

Sixteen women with advanced ovarian cancer participated (mean age = 52.3 ± 9.1 years, range = 38–69 years; Table 1). All had received platinum‐based chemotherapy; 56% received targeted therapy; 63% underwent surgery. Educational backgrounds: 31% primary school or below; 19% bachelor's degree or above. Employment status: 62.5% unemployed, 31.25% on leave, 6.25% currently working.

Findings reveal a triphasic psychosocial adjustment process organized around three core themes mapped to Leventhal's Common‐Sense Model (CSM) of self‐regulation (Figure 1): *Cognitive Reappraisal of Chronicity*, *Therapeutic Alliance as Hope Anchor*, and *Strategic Information Engagement* (Table 2). These interact dynamically to generate the trajectory from threat appraisal to adaptive hope. Sections below present participant narratives organized by theme.

**TABLE 2:** The example of analytical units, codes, sub‐theme and theme.

Themes	Sub‐themes	Categories
1. Cognitive reappraisal of chronicity	1.1 Therapeutic advances as a foundation for reappraisal	Treatment advances transform ovarian cancer from acute illness to manageable chronic condition Patients utilize medical analogies and visual metaphors to restructure illness identity Chronic illness mindset proves fragile; reverts when treatment effectiveness diminishes
	1.2 Temporality reconstruction: From survival countdown to living milestones Supporting narratives	Stable disease patients (≥ 18 months, *n* = 12/16) replace survival timelines with personal life milestones Improved psychological adaptation: Reduced mortality preoccupation, increased daily engagement Platinum‐resistant cases revert to acute survival‐countdown framing
	1.3 Information filtering and selective attention	Long‐term survivors (75%) actively filter illness information, prioritizing actionable biomarkers Selective attention evolves from broad post‐diagnosis data‐seeking to curated treatment‐specific updates Strategic information management reduces emotional distress and reinforces chronic illness frame
	1.4 Body‐disease relationship Reconfiguration	Phase 1 (0–3 months): Initial bodily betrayal, acute threat appraisal, mortality salience Phase 2 (4–10 months): Practical adaptations, initial therapeutic alliance formation Phase 3 (11+ months): Disease integration into self‐concept, stable chronic illness identity
2. Therapeutic alliance as hope anchor	2.1 Intentional shared decision‐making	Voice as validation of personhood and dignity during clinical encounters Fluid decision‐making adapting to fluctuating cognitive and emotional capacity Co‐creation of care plans valuing patient lived experience as clinical expertise
	2.2 Emotional attunement in clinical encounters	Nonverbal sensitivity and perceptive silence during clinical interactions Emotional mirroring validating unspoken fears and relational needs Trauma‐informed flexibility adapting to shifting emotional and psychological needs
	2.3 Continuity of care as hope reinforcement	Narrative coherence: “Being known” by care team as antidote to illness fragmentation Emotional anchoring: Longitudinal familiarity as buffer against uncertainty Anticipatory partnership: Proactive care planning and relational consistency enabling hope scaffold
	2.4 Honest prognostic communication framed with hope	Accurate medical information delivery without therapeutic nihilism or false reassurance Identification and emphasis of actionable aspects of care and treatment options Acknowledgment of uncertainties in disease trajectories and treatment responses Co‐construction of realistic, value‐aligned hope with individual patients
	2.5 Family support as mediating resource in hope maintenance	Family‐mediated illness identity reinforcement: Dyadic cognitive reappraisal of chronicity; family members reinforce or destabilize chronic illness frame through validating/invalidating responses Family as external cognitive scaffolding: Milestone documentation and temporal anchoring; family members maintain records of clinical outcomes and treatment milestones when patient attentional capacity is compromised Protective information mediation: Family gatekeeping and selective information filtering; active management of information flow to protect psychological equilibrium; variable outcomes dependent on coordination between family and clinical team Risk of uncoordinated communication: Unintended disclosure of prognostic information causing cognitive disruption and hope collapse; highlighting relational complexity in family‐mediated support systems
3. Strategic information engagement	3.1 Dignity through voice	Therapeutic listening establishes and maintains personal dignity during clinical encounters Clinician questioning transforms patient experience from passive recipient to active partner in care Recognition of patient experiential knowledge (symptom reports, treatment side effects, lived experience) as legitimate clinical data
	3.2 Dynamic flexibility	Illness dynamism requires agile, responsive communication strategies across treatment trajectory Real‐time communication shifts adapt to changing patient priorities and emotional capacity Flexibility strengthens therapeutic alliance and maintains engagement across phases of uncertainty
	3.3 Experiential validation	Systematic integration of patient‐reported symptoms into clinical decision‐making processes Treatment crises become “crisis insights” informing collaborative problem‐solving and care adaptation Co‐owned care solutions building patient trust, treatment adherence, and sense of agency

### 3.1 Cognitive Reappraisal of Chronicity (CSM Dimensions: Illness Identity, Timeline, Consequences)

This theme captures how patients progressively reconceptualized ovarian cancer from an acute, life‐threatening crisis into a manageable chronic condition—the primary site of illness identity transformation enabling sustained adaptive hope.

#### 3.1.1 Treatment Advances as Foundation for Cognitive Restructuring (CSM Dimension: Illness Identity)

Patients receiving targeted therapy actively drew on biomedical evidence—particularly scan results confirming disease stability—as the foundation for reconceptualizing their illness identity. The “stable disease” moment emerged as a critical cognitive threshold, triggering reframing from “terminal condition” to “manageable chronic illness”:

When my doctor showed me the scans after six months of olaparib treatment and said ‘stable disease,’ I realized—it's like my sister's diabetes. We can't cure it, but we can manage it. Those pills are now my insulin.

(P7, 54 years old)

This spontaneous biomedical analogy illustrates how patients mobilized familiar chronic illness frameworks to restructure catastrophic illness identity. Illness identity restructuring proved fragile under disease progression:

Don't call this a ‘chronic illness.’ Living with chronic cancer creates a persistent sense of vulnerability—like waiting for a storm that may or may not come. Each scan is like waiting for the next explosion.

(P4, 47 years old)

P4's platinum‐resistant status reveals that cognitive reappraisal is contingent on disease trajectory. Illness identity is not a permanently achieved state but a dynamically maintained construction, vulnerable to disruption at critical transition points.

#### 3.1.2 Temporal Restructuring: From Survival Countdown to Life Milestones (CSM Dimension: Timeline)

Patients reporting disease stability lasting ≥ 18 months demonstrated a discernible shift in temporal orientation—moving from survival countdowns toward meaningful life milestones (family events, personal projects). This shift accompanied improved psychological adaptation: reduced mortality preoccupation, increased daily engagement, and re‐emergence of future‐oriented thinking. In contrast, platinum‐resistant cases reverted to acute crisis perception:

The doctor said 'median survival is 3 years.' I marked it on my calendar—June 2025. This isn't a chronic illness; it's a countdown.

(P2, 62 years old)

#### 3.1.3 Information Filtering and Selective Attention (CSM Dimension: Consequences)

Long‐term survivors (*n* = 12/16) demonstrated strategic information management, prioritizing actionable biomarkers over distressing epidemiological statistics. This selective engagement evolved from broad‐based information seeking post‐diagnosis to increasingly selective curation of treatment‐specific updates:

In the beginning I read everything—survival rates, recurrence statistics. It made me sick with fear. Now I only look at my own numbers. My CA‐125, my scan results. Not other people's odds.

(P6, 51 years old)

This learned coping strategy helped manage emotional distress and reinforced the chronic illness frame by insulating it from threatening aggregate‐level data. As illustrated in Figure 1, information filtering functions as a feedback loop: illness identity restructuring enables selective engagement, which reinforces the protective chronic illness frame.

#### 3.1.4 Body‐Disease Relationship Reconfiguration (CSM Dimensions: Identity, Consequences)

Alongside cognitive reappraisal of illness identity, patients demonstrated progressive reconfiguration of their relationship with disease‐affected body across three phases, mirroring the broader adjustment trajectory.

##### 3.1.4.1 Phase 1—Initial Bodily Betrayal (0–3 Months)

Characterized by profound alienation from body, wherein physical sensations were interpreted exclusively through the lens of disease threat. Patients described visceral fear at bodily changes, experiencing the body as adversarial agent rather than extension of self:

My body became my enemy. Every ache, every numbness—I thought, 'Is it spreading? Is it worse?' I couldn't trust what my body was telling me.

(P11, 42 years)

Bodily symptoms triggered catastrophic threat appraisal, consuming cognitive capacity and exacerbating mortality salience characteristic of Phase 1 crisis cognition.

##### 3.1.4.2 Phase 2—Practical Adaptation (4–10 Months)

As treatment continuity emerged and therapeutic alliances deepened, patients transitioned from bodily rejection to pragmatic negotiation with physical limitation. Rather than interpreting bodily changes purely as pathological, they developed functional classification systems:

My hands go numb from the chemo. I used to panic. Now I adjust—different ways to hold things, different schedules for when it's worst. I'm managing the body, not fighting it.

(P8, 42 years)

This shift reflected nascent integration: bodily experience became knowable and manageable through active adaptation, paralleling concurrent cognitive reappraisal.

##### 3.1.4.3 Phase 3—Disease Integration (11+ Months)

Among patients achieving disease stability (*n* = 12/16), bodily experience became fully integrated into revised illness identity. Rather than interpreting bodily cues (fatigue, neuropathy, appetite changes) as harbingers of progression, patients reframed them as markers of ongoing treatment necessity:

I know my body now. This numbness means the drug is working. The fatigue is part of the deal. It's not my body attacking me—we're partners in this fight.

(P13, 38 years)

Notably, platinum‐resistant cases (*n* = 2) demonstrated reversal of this integration upon treatment failure, reverting to Phase 1 bodily alienation and confirming that embodied chronicity is dynamically maintained rather than permanently achieved.

### 3.2 Therapeutic Alliance as Hope Anchor (CSM Dimension: Controllability/Curability)

The quality of the patient–clinician relationship functioned as the relational scaffold enabling cognitive reappraisal. Therapeutic alliance was not described as a separate source of hope but as the relational condition making cognitive reappraisal possible—the mechanism linking “Therapeutic Alliance as Hope Anchor” to “Cognitive Reappraisal of Chronicity” in Figure 1.

#### 3.2.1 Intentional Shared Decision‐Making (CSM Dimension: Controllability)

Participation in treatment decisions provided existential agency that reinforced perceived controllability:

Having my voice heard in treatment planning isn't just about choice—it's about dignity. When my oncologist asks, 'What matters most to you right now?' I feel like a partner, not just a patient.

(P5, 60 years old)

Shared decision‐making positioned patients as “active managers” rather than passive recipients, directly supporting the chronic illness identity:

When side effects became severe, my doctor involved me in modifying the treatment plan. That respect for my experience made me trust the process more—and made me feel like this was something we could manage together, not something happening to me.

(P8, 42 years old)

#### 3.2.2 Emotional Attunement in Clinical Communication (CSM Dimension: Controllability/Emotional Representation)

Clinician emotional attunement moderated threat appraisal and enabled patients to tolerate uncertainty:

When I'm overwhelmed, my nurse knows to slow down and just sit with me. That attunement matters more than any pamphlet or procedure.

(P13, 38 years old)

Technical excellence isn't enough. My doctor picks up on my unspoken fears. That emotional awareness makes difficult conversations possible.

(P15, 69 years old)

Emotional attunement functioned as an external regulatory resource that patients internalized, building emotional tolerance for uncertainty that sustained adaptive hope.

#### 3.2.3 Continuity of Care as Hope Reinforcement (CSM Dimension: Controllability/Timeline)

Team consistency provided temporal scaffolding reinforcing milestone‐based temporal orientation:

My care team knows my story—not just my chart. They remember what worked before, what didn't, and why. That history builds trust.

(P15, 69 years old)

Having the same care team through everything—they're like an anchor in rough seas. They know when I'm struggling before I say it, and they've seen me bounce back before. That steady presence helps me believe I can get through tough times again.

(P13, 38 years old)

The care team's longitudinal presence enabled patients to perceive their illness timeline as accumulating resilience rather than counting down toward death.

#### 3.2.4 Hope‐Framed Honest Prognostic Communication (CSM Dimension: Controllability/Emotional Representation)

Patients valued communication maintaining prognostic accuracy while preserving cognitive space for hope:

My doctor is straightforward about my condition, but always focuses on what we can do. She doesn't sugarcoat things, but she also doesn't make me feel like giving up. That balance helps me face each day with clear eyes and an open heart.

(P7, 54 years old)

Actionable framing of clinical information sustained controllability perceptions:

They always help me see what I can actually do—whether it's adjusting my medications, staying active, or connecting with support groups. Having clear next steps makes me feel less overwhelmed and more in charge of my journey.

(P12, 61 years old)

Explicit acknowledgment of uncertainty paradoxically reinforced adaptive hope by modeling a stance compatible with the chronic illness frame:

My team is honest about what they don't know. Instead of pretending to have all the answers, they help me understand the range of possibilities. Somehow, that makes the uncertainty easier to bear.

(P11, 42 years old)

#### 3.2.5 Family Support as a Mediating Resource in Illness Cognition and Hope Maintenance

Family involvement facilitated cognitive reappraisal, temporal orientation, and information management in ways intersecting with hope maintenance. Three functional dimensions were identified.

##### 3.2.5.1 Co‐Construction of Chronic Illness Identity

Illness identity reappraisal appeared to function as a dyadic process, with family members serving as reinforcing or destabilizing agents:

Before, every time I said it was like diabetes, he would get sad. After the doctor explained it to him, he started saying it too. That's when I really believed it myself.

(P7)

I had convinced myself this was manageable. But every time she cried, I forgot everything I had been told.

(P12)

##### 3.2.5.2 Externalized Milestone Tracking and Temporal Anchoring

Family members provided external cognitive scaffolding compensating for patients' attentional limitations:

She writes down every good result. When I feel like giving up, she reads them back to me. It is not just support—she is the memory I cannot keep myself.

(P3)

I cannot see ahead. But he makes a mark every time. That is how I know I am still moving.

(P9)

##### 3.2.5.3 Selective Information Mediation and Protective Filtering

Family members actively managed clinical information flow to protect psychological equilibrium:

He reads everything first. Then he tells me what I need to know. I used to want to know everything. Now I trust him to filter it.

(P5)

My family talked to the doctor together. They decided together what to tell me, how to say it. I know some people would not like this. For me, it was protection.

(P14)

##### 3.2.5.4 Risk of Uncoordinated Family Communication

However, uncoordinated information mediation carried risk:

She didn't mean to say it. But once I heard it, I couldn't unhear it. Everything I had built up just collapsed.

(P11)

Unintended disclosure of prognostic information caused abrupt cognitive disruption, illustrating that family support systems require coordination with clinical communication to sustain hope effectively.

### 3.3 Strategic Information Management (CSM Dimensions: Cause, Controllability)

Strategic information engagement describes patients' active, self‐directed regulation of illness‐related information as cognitive coping mechanism. In the CSM framework (Figure 1), it represents the behavioral output of the two preceding themes: cognitive reappraisal establishes the illness framework to be protected, and therapeutic alliance provides relational security enabling selective rather than avoidant information engagement. This theme engages CSM dimensions of Cause and Controllability.

#### 3.3.1 Dignity Through Voice and Experiential Validation (CSM Dimension: Illness Identity/Controllability)

Patients described recognition of their experiential knowledge by clinicians as foundational to information engagement:

Being heard in treatment planning transcends mere clinical decision‐making—it's about maintaining personal dignity during illness. The simple yet powerful question 'What matters most to you right now?' transforms the patient experience from being a passive recipient to an active partner in care.

(P4, 60 years old)

Therapeutic listening and clinician questioning transformed patients' relationship to illness information, positioning them as expert partners in decision‐making rather than passive recipients of medical facts.

#### 3.3.2 Dynamic Flexibility Across Treatment Phases (CSM Dimension: Controllability)

The fluctuating nature of illness required patients to adapt their information needs across treatment phases—a flexibility that was only possible when the therapeutic alliance supported variability in communication style:

My needs fluctuate throughout my treatment journey, requiring healthcare providers to demonstrate flexibility in their communication and decision‐making styles. This responsiveness to changing patient preferences strengthens the therapeutic alliance.

(P1, 45 years old)

Real‐time communication shifts sustained patient‐centered priorities across phases of medical uncertainty, preventing information dysregulation.

#### 3.3.3 Experiential Validation and Collaborative Problem‐Solving (CSM Dimension: Consequences/Controllability)

Integrating patient‐reported symptom experience into clinical decision‐making transformed information exchange into a collaborative process, reinforcing the chronic illness management frame:

When my pain became unbearable after chemo, my oncologist didn't just adjust my medications—she sat down and asked, 'What specifically feels worse? Should we slow down or try a different approach?' That conversation made me believe we were truly partners in this.

(P7, 54 years old)

Treatment crises became “crisis insights” informing collaborative adaptation, building patient trust and reinforcing chronic illness management frame.

**FIGURE 2:**
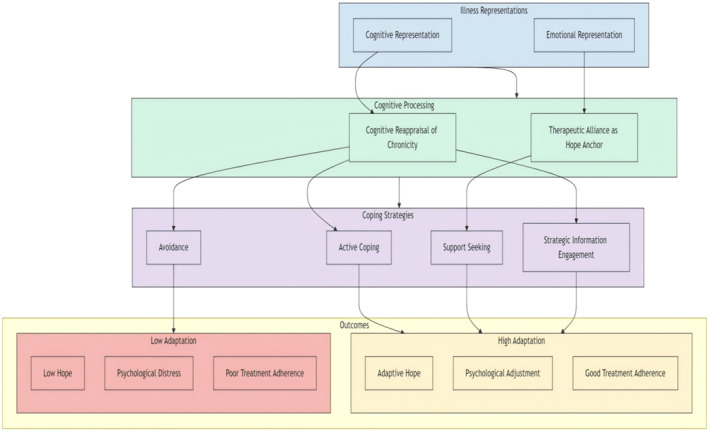
Illness cognition to hope maintenance pathway model in advanced ovarian cancer.

Taken together, the themes presented in Sections 3.1, 3.2, 3.3 describe the key cognitive, relational, and behavioral processes through which patients maintained hope during advanced ovarian cancer treatment. As summarized in Figure 1, these themes map onto core cognitive and emotional dimensions of Leventhal's Common‐Sense Model. When considered together over time, they also informed the integrative pathway shown in Figure 2, which illustrates how these processes unfolded across the illness trajectory to support, or disrupt, adaptive hope.

### 3.4 Integrative Framework: Triphasic Chronic Illness Adjustment Model (Figure 2)

Building on the thematic findings presented above, Figure 2 synthesizes the data into an integrative pathway model of hope maintenance in advanced ovarian cancer. Whereas Figure 1 shows how the themes correspond to CSM dimensions, Figure 2 illustrates how these same processes unfold over time as a dynamic adjustment trajectory. Across the dataset, adaptive hope emerged through the interaction of cognitive reappraisal, therapeutic alliance, and strategic information management.

#### 3.4.1 The Triphasic Adjustment Process

Phase 1 was characterized by crisis cognition, typically occurring in the early months following diagnosis or recurrence. During this period, illness identity was dominated by acute threat appraisal, mortality salience, and a sense of bodily betrayal. Temporal orientation was often framed as a survival countdown, and hope remained fragile and contingent on immediate clinical outcomes. Therapeutic alliance was still developing, and information engagement was often broad, urgent, and dysregulating.

Phase 2 involved cognitive negotiation. As treatment responses emerged and therapeutic alliances deepened, patients began to selectively reappraise illness representations, particularly in relation to identity, timeline, and controllability. For many participants, the first “stable disease” scan functioned as a clinical anchor that supported a shift from an acute crisis frame toward a chronic illness frame. Hope became increasingly grounded in relational continuity, functional stability, and the possibility of living forward despite uncertainty.

Phase 3 reflected adaptive integration. Patients who achieved relative disease stability described a more durable chronic illness identity, a milestone‐based temporal orientation, and more proactive forms of selective information engagement. In this phase, hope was reconstituted as a forward‐looking and action‐oriented stance that remained compatible with ongoing medical uncertainty rather than dependent on cure.

This trajectory was neither linear nor universal. In platinum‐resistant cases, participants frequently reverted to Phase 1 crisis cognition, indicating that adaptive integration was dynamically maintained rather than permanently secured. This pattern underscores the clinical importance of renewed support at moments of disease progression.

#### 3.4.2 Link to the CSM Framework

The pathway model shown in Figure 2 is grounded in the CSM structure outlined in Figure 1. Cognitive reappraisal of chronicity primarily involved shifts in illness identity, timeline, and consequences; therapeutic alliance supported emotional regulation and perceived controllability; and strategic information management reflected active regulation of cause‐ and control‐related information. Rather than presenting a separate framework, Figure 2 extends Figure 1 by showing how these CSM‐linked processes unfolded over time and converged in either adaptive or maladaptive outcomes.

### 3.5 Age‐Related Patterns in Hope Maintenance

Younger patients (≤ 45; *n* = 5)tended to articulate hope through future‐oriented goals such as parenting and professional roles. This group appeared to experience more acute existential distress and more effortful Phase 2 negotiation. Once achieved, chronic illness reappraisal became anchored to concrete life milestones, suggesting that hope maintenance in younger patients was closely tied to role continuity.

Patients aged (46–60; *n* = 6)expressed a more pragmatic form of hope that was anchored to disease control and everyday stability. Compared with younger patients, this group demonstrated a more moderate level of existential distress, with greater emphasis on functional independence and daily stability rather than role disruption. Their Phase 2 negotiation appeared less intense and involved a more straightforward process of cognitive reappraisal.

Older patients (≥ 60; *n* = 5) drew more frequently on prior illness experiences as cognitive resources and appeared to reach Phase 3 integration with less Phase 2 negotiation. In this group, hope was more often anchored to meaning‐making and existential acceptance rather than future role restoration.

## 4 Discussions

### 4.1 Main Findings

This study identified three interdependent mechanisms structuring adaptive hope in advanced ovarian cancer: illness identity reappraisal, therapeutic alliance as relational scaffold, and strategic information management. Adaptive hope emerged not as a static trait but as an actively maintained cognitive construction requiring ongoing relational support.

#### 4.1.1 Illness Identity Reappraisal

Transitioned patients from “acute crisis” to “manageable chronic condition,” catalyzed by clinical anchors such as the first “stable disease” scan. P7's analogy—“It's like my sister's diabetes… Those pills are now my insulin”—illustrates how biomedical evidence restructured illness identity. Critically, this reappraisal proved contingent on disease trajectory: platinum‐resistant cases reverted to crisis cognition (P4: “Each scan is like waiting for the next explosion”), confirming that illness identity is dynamically maintained rather than permanently secured.

#### 4.1.2 Therapeutic Alliance

Functioned as the relational precondition enabling cognitive reappraisal. Shared decision‐making restored existential agency (P5: “it's about dignity”), while emotional attunement co‐regulated threat appraisal. Continuity of care sustained milestone‐based temporal orientation (P13: “They've seen me bounce back before”).

#### 4.1.3 Strategic Information Management

Constituted behavioral protection of cognitive frameworks. Patients actively regulated their information environment (P6: “Now I only look at my own numbers… Not other people's odds”), reinforcing cognitive reappraisal through iterative feedback loops.

Together, these three mechanisms generate the triphasic trajectory—crisis cognition → cognitive negotiation → adaptive integration—confirming CSM's proposition that parallel cognitive and emotional processing jointly shape behavioral outcomes.

### 4.2 Theoretical Implications for the Common‐Sense Model

The findings presented in Figure 2 extend Leventhal's Common‐Sense Model by suggesting that adaptation in advanced ovarian cancer is not adequately captured as a linear sequence from illness representation to coping and outcomes. Instead, our data indicate a bidirectional and reinforcing relationship between cognitive reappraisal and behavioral information regulation. Once patients begin to reinterpret the illness as manageable rather than acutely catastrophic, they selectively regulate their information environment in ways that protect this emerging cognitive frame. In turn, this strategic information management helps stabilize the reappraisal process and supports the maintenance of adaptive hope.

This interpretation is particularly relevant in advanced ovarian cancer, where uncertainty is prolonged and disease stability is often temporary rather than curative. Within this context, hope was sustained not simply through positive appraisal, but through the interaction of cognitive, relational, and behavioral processes [31]. The model therefore highlights the importance of understanding adaptation as dynamically maintained and vulnerable to disruption, especially at moments of progression or recurrence.

### 4.3 Cognitive Processing Mechanisms

Cognitive reappraisal (reframing cancer as manageable rather than catastrophic) aligns with Audulv et al.'s findings on cognitive reframing facilitating psychological adjustment in cancer [32]. Therapeutic alliance emerged as a powerful anchor for hope, with patients referencing clinician communications as touchstones during uncertainty—consistent with Hancock et al.'s research on prognostic discussions balancing honesty with hope [33].

### 4.4 Coping Strategies and Adaptation Outcomes

Active coping, support‐seeking, and strategic information engagement were associated with high adaptation outcomes (adaptive hope, psychological adjustment, treatment adherence), consistent with Stanton et al., [34]. Conversely, avoidance coping predicted low outcomes, aligning with Roesch et al.'s research [35].

A novel finding was strategic information engagement as adaptive coping rather than problematic avoidance. While similar patterns appear in breast cancer [36], this study extends findings to advanced ovarian cancer under targeted therapy, where selective engagement protects hard‐won chronic illness identity without compromising decision‐making access [37].

### 4.5 Important Moderators: Age, Culture, and Disease Heterogeneity

#### 4.5.1 Age‐Related Patterns

These preliminary observations suggest age‐sensitive communication calibration may enhance clinical relevance. Future age‐stratified studies are warranted.

#### 4.5.2 Cultural Context

“Distributed health literacy”—where family members actively participate in information filtering—represents an important contextual factor in collectivist settings [38]. This contrasts with Western models emphasizing individual autonomy, suggesting culturally congruent interventions should integrate rather than minimize family participation [39].

#### 4.5.3 Disease Subtype Considerations

The majority (*n* = 12, 75%) were HGSOC; others included endometrioid, clear cell, and low‐grade serous. The insulin‐diabetes analogy anchoring cognitive reappraisal was explicitly tied to PARP inhibitor maintenance—predominantly available to HGSOC patients with BRCA/HRD positivity. Patients lacking targetable mutations may have fewer “chronic management” analogies. Low‐grade serous patients showed more protracted Phase 2 negotiation. Future histologically stratified studies are needed; interventions should be understood as most directly applicable to HGSOC/PARP inhibitor‐eligible populations.

### 4.6 Comparison With Existing Literature

#### 4.6.1 Cognitive Representation Dimensions

Our findings support Leventhal et al.'s proposition about illness identity influencing coping strategies [4], identifying a unique “chronicization” reframing pattern specific to advanced ovarian cancer. Unlike the “cure narrative” in early‐stage breast cancer patients [40], our participants viewed PARP inhibitors as “insulin‐like long‐term management medications,” establishing more realistic illness identities.

#### 4.6.2 Timeline Perception

Our “milestone‐based tracking” strategy echoes Michael et al.'s concept of “temporal anchoring” [41], but demonstrates greater adaptability in the advanced ovarian cancer context, transforming treatment cycles into concrete steps against uncertainty. This extends Chan's work on treatment control beliefs among Chinese cancer patients [42], highlighting how PARP inhibitor mechanism understanding specifically strengthens internal control, differing from Western research on general control concepts.

#### 4.6.3 Emotional‐Cognitive Representation Transformation

Our study addresses a less explored CSM dimension: how threat appraisal transforms into hope adaptation through family mediation [43]. This is consistent with Chan et al.'s research on Chinese families' central role in cancer management [42], but articulates specific mediation mechanisms. Unlike Hagger et al.'s emphasis on “autonomous control” in prostate cancer research [44], our findings demonstrate that within Chinese cultural contexts, family involvement enhances rather than diminishes disease management capacity.

### 4.7 Clinical Implications and Evidence‐Based Approaches

*Methodological Note:* This study was observational, examining how naturally occurring clinical interactions relate to illness cognition within CSM framework. This enables identification of practices aligning with adaptive hope maintenance warranting formal testing.

Language‐based reframing interventions in chronic illness management (lung cancer [45], multiple myeloma [46]) show that shifting from “terminal” to “chronic disease management” framing improves psychological adjustment and adherence [47]. In advanced ovarian cancer, the first “stable disease” scan represents a clinically opportune moment for chronicity framing, with PARP inhibitor mechanism providing concrete anchors.

#### 4.7.1 Recommended Approach 1: Family‐Inclusive Illness Identity Reframing

In 68.8% of cases (*n* = 11/16), family members mediated clinical information, supporting cognitive reappraisal when family responses were validating. Family emotional incongruence undermined adjustment. *Recommended practice:* Involve primary caregivers in illness framing conversations while attending to individual variation in family involvement preferences. Clinical training should address family emotional dynamics while respecting autonomy.

#### 4.7.2 Recommended Approach 2: Milestone Tracking Programs

Milestone‐based temporal reorientation reduces uncertainty‐related distress in breast and colorectal cancer [48]. In advanced ovarian cancer, tracking proved meaningful when anchored to treatment cycles rather than calendar time. Family members contributed by maintaining shared records as external memory aids. Among family co‐constructed tracking participants (*n* = 9), temporal orientation appeared more stable across the triphasic trajectory versus independent tracking (*n* = 7), though formal validation is required. *Recommended development:* Design family‐inclusive milestone tools calibrated to treatment cycles, evaluated through prospective trials with standardized measures.

#### 4.7.3 Recommended Approach 3: Family Information Mediation Training

Family‐based interventions in Chinese oncology reduce caregiver burden [49]. Our data suggest a specific mechanism—family involvement in selective information mediation—requiring replication. Across 81.3% of participants (*n* = 13/16), family members mediated information prior to patient disclosure, consistent with collectivist norms [4, 50]. Family information mediation protects psychological equilibrium but risks disrupting coping frameworks if uncoordinated. *Recommended practice:* Develop clinical training programs addressing family information management as a distinct competency, helping clinicians navigate family dynamics while respecting cultural norms, maintaining patient autonomy, and coordinating disclosure across care teams.

These three approaches address cognitive, temporal, and relational dimensions of hope maintenance. We recognize their preliminary nature and call for rigorous evaluation through randomized controlled trials with attention to cultural adaptation fidelity [51].

### 4.8 Study Limitations

Several methodological and sample‐related limitations substantially constrain the interpretive scope of this study.

#### 4.8.1 Sample Size and Transferability

The most significant limitation is the small sample (*n* = 16) from a single tertiary center in southern China. While appropriate for phenomenological framework development, the sample size is insufficient to support confident generalization of coping patterns or intervention recommendations to other ovarian cancer populations.

#### 4.8.2 Absence of Age‐Stratified Design

Although post‐hoc thematic examination identified potentially meaningful age‐related differences in hope maintenance mechanisms, the study was not designed with age stratification as a sampling criterion, and subgroup sizes are too small to support robust age‐stratified analysis. The age‐related observations reported here should be considered hypothesis‐generating rather than confirmatory, and future studies should employ intentional age‐stratified sampling to examine age as a potential moderator of illness cognition trajectories.

#### 4.8.3 Histological Subtype Homogeneity

The sample was predominantly composed of high‐grade serous ovarian carcinoma patients (*n* = 12, 75%), reflecting the expected case‐mix at a tertiary referral center but limiting the generalizability of findings to other histological subtypes. The chronicization reappraisal mechanism identified in this study is partly contingent on PARP inhibitor eligibility, which varies substantially by subtype. Future research should employ histologically stratified designs to enable systematic comparison of illness cognition patterns across ovarian cancer subtypes.

#### 4.8.4 Absence of Disease Progression Events

Despite the 11‐month longitudinal follow‐up, data were collected at discrete interview timepoints rather than continuously, meaning the triphasic trajectory is reconstructed from interview accounts rather than directly observed in real time. Self‐report and retrospective recall may have introduced memory biases, and the absence of baseline psychological measures limits our ability to distinguish cognitive patterns that pre‐dated diagnosis from those that emerged in response to it.

#### 4.8.5 Recruitment Bias

Recruitment likely favored patients with stronger communication abilities and higher health literacy. Patients with severe psychological distress, communication difficulties, or lower literacy are systematically under‐represented, introducing optimistic bias. Generalizability to patients with lower communication capacity remains unclear.

#### 4.8.6 Temporal and Design Limitations

Despite the 11‐month longitudinal follow‐up, data were collected at discrete interview timepoints rather than continuously, meaning the triphasic trajectory is reconstructed from interview accounts rather than directly observed in real time. Self‐report and retrospective recall may have introduced memory biases, and the absence of baseline psychological measures limits our ability to distinguish cognitive patterns that pre‐dated diagnosis from those that emerged in response to it.

#### 4.8.7 Cultural and Institutional Specificity

The family‐centric decision‐making and information filtering documented in this study reflects a culturally specific configuration that may not generalize to individualistic healthcare contexts. Similarly, the tertiary setting's advanced targeted therapy capabilities may have supported the adaptive coping patterns observed in ways that would not replicate in resource‐limited settings.

#### 4.8.8 Methodological Reflexivity

Inter‐coder reliability was established through systematic independent coding and consensus‐based resolution of discrepancies, but qualitative interpretative processes remain susceptible to researcher perspective despite reflexivity efforts. The theoretical framework (Leventhal's CSM) may have shaped analytic attention in ways that privileged CSM‐consistent patterns over alternative explanatory structures.

Despite these limitations, the findings contribute a theoretically grounded, data‐anchored conceptual framework for understanding illness cognition and hope maintenance in advanced ovarian cancer—one that, pending prospective validation, may inform targeted psychosocial interventions in this population.

### 4.9 Research Contributions and Future Directions

This study makes several important contributions at the theoretical, clinical, and integrative levels. Theoretically, it establishes a three‐tier illness cognition model grounded in the Common‐Sense Model (CSM), articulating a bidirectional feedback process between cognitive representations and behavioral information regulation, thereby extending standard CSM theory. Clinically, it proposes hope‐framed communication strategies for family‐mediated healthcare, including selective transparency practices that honor cultural norms while preserving patient autonomy. In addition, the study advances the integration of psychosocial support into oncology care by identifying the relational and cognitive mechanisms through which therapeutic interactions contribute to hope maintenance and treatment adherence.

Future research should focus on prospective quantitative validation using a sample of at least 200 participants to test the predictive validity of TCIAM. Further studies should also examine age as a potential moderator through age‐stratified analyses and incorporate longitudinal follow‐up to capture recurrence‐related disruption over time. Randomized trials are needed to evaluate the effectiveness of the recommended interventions, while cross‐subtype comparative analyses could clarify whether these patterns vary across cancer subtypes. Cultural adaptation studies conducted in Western contexts would also help assess the transferability of the model, and implementation science research is needed to support clinical translation.

Several clinical recommendations also emerge from these findings. Hope‐framed communication should be implemented at stable disease milestones, and emotional attunement and shared decision‐making should be treated as core clinical competencies. Psychosocial assessment should be integrated into standard care protocols. At the systems level, healthcare services should expand insurance coverage for psychological support, incorporate validated psychological metrics into quality assessment frameworks, and develop training programs focused on cancer‐specific psychological care.

### 4.10 Conclusion

This phenomenological study identified a theoretically grounded framework for understanding illness cognition and hope maintenance in advanced ovarian cancer—a population for whom targeted therapies create new chronic management possibilities yet psychosocial infrastructure remains underdeveloped.

The key insight is that adaptive hope is an actively maintained construction requiring: (1) cognitive reappraisal scaffolded by clinical evidence, (2) therapeutic alliance providing relational foundation, and (3) strategic information management protecting cognitive frameworks. This framework identifies specific intervention targets through which clinical interactions translate into better psychological outcomes and treatment adherence. Pending prospective validation and implementation in diverse settings, these findings may inform targeted psychosocial interventions enhancing hope maintenance in this population.

## Author Contributions

**Y.L.:** conceptualization, formal analysis, investigation, methodology, writing – original draft, writing – review and editing. **P.J.:** conceptualization, formal analysis, funding acquisition, investigation, methodology, supervision. **C.C.Z.:** formal analysis, investigation, methodology. **C.L.W.:** formal analysis, writing – review and editing. **F.H.:** formal analysis, methodology. **Y.F.W.:** conceptualization. **H.X.:** conceptualization. **Y.H.S.:** conceptualization. **C.R.W.:** methodology. All authors agree to be accountable for the content of the work.

## Funding

This work was supported by Shenzhen Science, Technology and Innovation Commission (NO: JCYJ20240813115013017); Shenzhen Health Economics Society (NO: 202507).

## Supporting information


Supporting Information S1

Supporting Information S2

## Data Availability

The authors have nothing to report.
