# Supplementary material for: Navigating Hope and Illness Cognition in Advanced Ovarian Cancer Patients: A CSM‐Based Phenomenological Study
Source: Psychooncology. 2026 Jun 12;35(6):e70506. doi: 10.1002/pon.70506 (PMC13263711; doi:10.1002/pon.70506)
Supplement: Supplementary file 1 — Supporting Information S1 [file PON-35-e70506-s001.pdf]

伦理审查批件

批件号：SFYLS[2023]039

项目名称	妇科癌症患者疾病获益感的纵向研究		
项目来源	研究者发起研究		
项目负责人	何芳	所在科室	妇科
申办方/资助方	NA	CRO 公司	NA
审查文件	初始审查申请表<br>附审查文件清单		
审查类别	☑ 初始审查<br>☐ 年度/定期跟踪审查报告<br>☐ 结题审查<br>☐ 严重不良事件事件/非预期事件报告审查<br>☐ 其它：	☐ 复审<br>☐ 修正案审查<br>☐ 暂停/终止已批准研究审查<br>☐ 不依从/违背方案事件审查	
审查方式	☐会议审查 ☑简易审查 ☐紧急会议审查		
会议日期	NA	审查会议地点	NA
投票结果	共有委员 NA 名，实到 NA 名，投票 NA 名，回避 NA 名		
	同意 NA 票	修改后同意 NA 票	
	不同意 NA 票	暂停或者终止研究 NA 票	
审查意见	审查决定：同意<br>根据《涉及人的生物医学研究伦理审查办法》（2016 年）、《药物临床试验质量管理规范》（2003 年）、《药物临床试验伦理审查工作指导原则》（2010 年）、WMA《赫尔辛基宣言》和 CIOMS《涉及人的健康相关研究国际伦理指南》的伦理原则。<br>经本伦理委员会审查同意按递交的研究方案、知情同意申请。开展本项研究。<br>注：<br>1、请遵循 GCP 原则、遵循伦理委员会同意的方案开展临床研究保护受试者的健康和权利。<br>2、对研究方案、知情同意书、招募材料等的任何修改请提交修正案审查申请。<br>3、发生 SAE 请及时提交严重不良事件报告。<br>4、如有不依从/违背方案的情况请及时提交违背方案报告。<br>5、请根据年度/定期跟踪审查频率及时提交研究进展报告。<br>6、暂停或终止临床研究请及时提交暂停/终止研究报告。<br>7、完成临床研究请提交结题报告。		
年度定期/跟踪审查频率	12 个月	批件有效期	1年<br>（请在 2024 年 10 月 9 日前提交跟踪审查申请）
联系人	戴宇婷	联系电话	82869849
主任委员（被授权人）签名	（签名）	深圳市妇幼保健院科研伦理委员会<br>2023 年 11 月 10 日	

深圳市妇幼保健院医学伦理委员会

扫描全能王 创建

科研伦理委员会伦理审查文件清单

批件号：SFYLS[2023]039

研究项目：
妇科癌症患者疾病获益感的纵向研究

审查文件清单：
（1）初始审查申请表
（2）项目材料诚信承诺书
（3）研究方案（版本号：1.0 版本日期：2023年6月30日）
（4）知情同意（版本号：1.0 版本日期：2023年6月30日 ）
（5）主要研究者履历
（6）组长研究单位伦理相关结果
（7）开题报告
（8）调查问卷（版本号：V1.0 版本日期：2023年6月30日）

深圳市妇幼保健院科研伦理委员会

2023年11月10日

扫描全能王 创建
