# Supplementary material for: Navigating Hope and Illness Cognition in Advanced Ovarian Cancer Patients: A CSM‐Based Phenomenological Study
Source: Psychooncology. 2026 Jun 12;35(6):e70506. doi: 10.1002/pon.70506 (PMC13263711; doi:10.1002/pon.70506)
Supplement: Supplementary file 2 — Supporting Information S2 [file PON-35-e70506-s002.pdf]

知情同意书

尊敬的就诊者：

我们将邀请您参与一项关于“希望与现实的认知调适历程：晚期卵巢癌患者疾病认知的现象学研究——基于CSM模型”的调查研究。在您决定是否参加这项研究前，请仔细阅读本知情同意书的全部内容，它可以帮助您了解本研究（或新技术）的目的、流程、期限以及参与研究后可能给您带来的收益、风险与不适。当研究人员向您说明和讨论知情同意书时，您可以随时提出疑问并要求研究人员予以解释。您可以与您的家人、朋友以及您的主治大夫讨论后再做决定。

项目基本情况

项目名称：希望与现实的认知调适历程：晚期卵巢癌患者疾病认知的现象学研究——基于 CSM 模型

项目来源：自选课题

研究机构：深圳市妇幼保健院

研究目的和意义

卵巢癌作为妇科恶性肿瘤病死率最高的肿瘤，严重地威胁着女性的健康。晚期卵巢癌患者在诊断、治疗及后续过程中会面对多种治疗决策，卵巢癌患者缺乏疾病概念状态，造成对疾病的认识有限。晚期卵巢癌患者如何描述他们意识到自己疾病的经历，可增强患者积极应对疾病和死亡威胁的信念。随着精准医疗的快速发展，综合治疗为晚期肺癌患者带来了新的希望，但疗效存在着个体差异，复发率高，预后差，使得希望破裂。因此，本研究基于 Parse 人类适转理论，探讨晚期卵巢癌综合治疗后患者疾病认知状态体验及希望，从而为后期临床制定针对性的护理干预措施提供现实依据。

研究对象

本研究计划招募 18 名受试者，其中：

纳入标准：①经病理诊断为卵巢肿瘤；②至少接受过 6 个疗程化疗，目前接受治疗；③年龄≥18 岁；④知晓自身病情诊断。

排除标准：①合并其他系统并发症，病情危重；②严重的精神、认知障碍；③语言沟通障碍。

③语言沟通障碍。

研究过程

以半结构化、面对面访谈的方式于受访者在院治疗期间收集受访者的资料。研究中的每个女性都被采访了两次；这第二次面试发生在第一次面试后的两到四周面试。每次采访时长60分钟，并且是数字化的 记录和专业转录。数据收集 第一次访谈侧重于患者对以下内容的理解，确诊后，对于疾病的心态是如何变化的，。 在第二次采访中，参与者被问到 类似的问题，以澄清某些不在第一次面试中明确或需要更多的探索。访谈结束后及时书写反思日记，文本资料整理后返回受访者处确认。

潜在风险与不适

本课题为采用问卷的重复测量调查研究，课题不需要收集研究对象的任何生物学标本，本研究不会对研究对象造成不良反应。研究过程中参与者可随时退出研究。

隐私保护

如果您决定参加本项研究，您参加试验及在试验中的个人资料均属保密。您的组织标本将以研究编号数字而非您的姓名加以标识。可以识别您身份的信息将不会透露给研究小组以外的成员，除非获得您的许可。所有的研究成员和研究申办方都被要求对您的身份保密。您的档案将保存在有锁的档案柜中，仅供研究人员查阅。为确保研究按照规定进行，必要时，政府管理部门或伦理审查委员会的成员按规定可以在研究单位查阅您的个人资料。这项研究结果发表时，将不会披露您个人的任何资料。

相关费用

此项目所需要的有关研究费用由课题组承担，您不需要负担研究过程本身所需要的的任何费用。本项目不会增加您的诊疗费用，您无需承担常规诊疗之外的任何费用。

参加本项目可能的获益

扫描全能王 创建

参与研究者可获得更多的医疗资源和帮助。如：获得更多的疾病教育、行为矫正和问题解决技术等。且您提供的宝贵资料将帮助科研及医务人员有效地了解疾病获益感的发展轨迹。

作为受试者，您有以下义务：

①真实、准确地回答或提供研究所需信息和内容；

②及时告知医生自己在本次研究期间所出现的任何不适；

③告诉研究人员自己是否参与其它研究或曾经参与其它研究。

您可以选择不参加本次研究，即便是参加研究后，您也可以随时要求退出本次研究，若您决定中途退出研究，请您主动告知研究人员，您不会因此受到歧视或报复，您的医疗待遇与权益也不会因此受到影响。

在参与研究的过程中，如果您需要进行其它治疗，或者您没有遵循既定研究计划，或者发生了与研究相关的损伤，研究人员可以终止您继续参与本次研究。

您可以随时了解与本研究有关的内容和研究进展，如果您有与本研究相关的问题，或您在研究过程中发生了任何不适与损伤，或有关于本项研究参与者权益方面的问题您可以通过手机电话13823316810与研究人员龙云联系。

****************** ↓知情同意书签字页↓ *******************

扫描全能王 创建

知情同意书签字页

受试者同意声明：

我已认真阅读本知情同意书的全部内容，研究人员已向我解释了本项研究，并且我有机会就此项研究与研究人员讨论并提出问题，我提出的所有问题都得到了满意的答复。

我知道参加本项研究可能产生的风险和受益，我决定参加此项研究是完全自愿的，我有权随时退出本项研究。

此项研究记录及我的医疗记录可能会被研究者授权的代表人、主管机构或与我参加的此项研究相关的伦理委员会查看。我允许这些人查阅我的医疗记录，并且我知道这些信息将被保密。

我同意参加此项研究。

受试者（或法定代理人）签名（正楷）：方[redacted]

联系电话：158[redacted]　日期：2023年 11月 23日

（注：如受试者无行为能力时需代理人签名）

研究者声明：

我将严格遵循临床研究相关法律法规及相关行为准备，保证受试者的医疗待遇和合法权利得到充分尊重，并将严格保护受试者隐私信息。

研究者签名（正楷）：龙云

联系电话：13823316810　日期：2023-11-23

扫描全能王 创建
